# Parasitoid vectors a plant pathogen, potentially diminishing the benefits it confers as a biological control agent

**DOI:** 10.1038/s42003-021-02851-2

**Published:** 2021-11-25

**Authors:** Chang-Fei Guo, Muhammad Z. Ahmed, Da Ou, Li-He Zhang, Zi-Tong Lu, Wen Sang, Cindy L. McKenzie, Robert G. Shatters, Bao-Li Qiu

**Affiliations:** 1 Key Laboratory of Bio-Pesticide Innovation and Application of Guangdong Province, South China Agricultural University, Guangzhou, 510640 China; 2 Guangdong Laboratory for Lingnan Modern Agriculture, Guangzhou, 510640 China; 3 Engineering Research Center of Biocontrol, Ministry of Education, Guangzhou, 510640 China; 4 Subtropical Insects and Horticulture Research Unit, Agricultural Research Service, USDA, Fort Pierce, FL 34945 USA; 5 College of Life Sciences, Chongqing Normal University, Chongqing, 401300 China

**Keywords:** Microbial ecology, Microbial communities, Entomology

## Abstract

Huanglongbing (HLB) is a destructive disease of citrus primarily transmitted by the Asian citrus psyllid (ACP). Biocontrol of ACP is an environmentally sustainable alternative to chemicals. However, the risk of parasitoid rational application in ACP biocontrol has never been evaluated. Here we show, the dominant parasitoid of ACP, *Tamarixia radiata*, can acquire the HLB pathogen *Candidatus* Liberibacter asiaticus (*C*Las) and transmit it horizontally when probing ACP nymphs. If these ACP nymphs survive the probing, develop to adults and move to healthy plants, *C*Las can be transmitted to citrus leaves during feeding. We illustrate the formerly unrecognized risk that a parasitoid can potentially serve as a phoretic vector of the pathogen transmitted by its host, thus potentially diminishing some of the benefits it confers via biocontrol. Our findings present a significant caution to the strategy of using parasitoids in orchards with different infection status of insect-vectored pathogens.

## Abstract (web summary)

Guo et al. demonstrate the possibility of a parasitoid to contract and transmit a bacterial pathogen via its insect host to the citrus plant it is used to protect. This reduces the efficacy of the biological control agent to protect the plant from ill effects.

## Introduction

Citrus Huanglongbing (HLB) or citrus greening has become one of the world’s most destructive diseases since its global spread during the last 20 years. It was first reported from southern China in 1919 and now is known to occur in approximately 40 different countries across Asia, Africa, Oceania, South and North America^1,2^. It has been estimated to have cost Florida’s economy over US$4.4 billion and resulted in over 8257 job losses since 2006^3^. The causative agents of HLB are three vector-borne α-proteobacteria, of which the most widespread and pervasive is *Candidatus* Liberibacter asiaticus (*C*Las), vectored by the Asian citrus psyllid (ACP), *Diaphorina citri* Kuwayama^1^. Therefore, effective control of ACP is a key component of *C*Las management globally.

Chemical control has been used aggressively to eliminate ACP from citrus orchards in many countries. However, the frequent use of insecticides has resulted in the evolution of insecticide resistance for ACP in many parts of the world, caused a decrease in the populations of ACP natural enemies, and raised concerns regarding human health and environmental sustainability as well as citrus production costs^4–6^. Thus, increasing effort has been placed on developing biological control solutions to ACP. Among the biological control agents of ACP, *Tamarixia radiata* (Waterston) is considered the dominant parasitoid globally^7,8^. For example, the parasitism of *T. radiata* on ACP was approximately 36% in summer and 46% during autumn in China^9^. It was observed to vary between 20 and 56% in Florida^10^, and averaged around 21% in southern California in autumn^11^. Pluke et al.^12^ also observed between 70 and 100% parasitism in Puerto Rico. *T. radiata* is a solitary, arrhenotokous ectoparasitoid, adult females of *T. radiata* feed on all the nymphal instars; however, they generally oviposit on the older nymphal instars (preferentially fourth and fifth instars)^13,14^. This parasitoid has been used for the biological control of ACP in many regions of the world, and one field investigation has successfully demonstrated suppression in the ACP population in the natural environment without applying insecticides^12^.

Prior studies have revealed two main routes for the increasing distribution of the *C*Las pathogen; grafting and ACP feeding between *C*Las infected and healthy but susceptible citrus plants. However, during biological control of ACP using *T. radiata*, we unexpectedly found that *T. radiata* can acquire *C*Las as nymphs and subsequent adults can transmit it to ACP nymphs. This observation raises the concern that such transmission of *C*Las by *T. radiata* could diminish the benefits of biological. In the current study, we evaluated the acquisition, persistence, and transmission of *C*Las pathogen in the *T. radiata*-ACP-citrus plant tritrophic system. We present a new insight into parasitoid-based biological control of ACP and reveal a new route of *C*Las transmission. The discovery that *Tamarixia* parasitoid can function as a vector of *C*Las creates an academic need to understand this impact on *C*Las transmission in the field. *Tamarixia* parasitoid is used in biological control to reduce the ACP population and thus reduce the transmission of *C*Las from infected to healthy citrus. The question now is raised: is the increased percentage of the ACP population that is infected with *C*Las, as a result of *Tamarixia* parasitoid transmission, significant enough to outweigh the benefit achieved by ACP population reduction through *Tamarixia* parasitism? Also, our findings demonstrate that the potential negative effect of parasitoid-based horizontal transmission of plant pathogens should carefully appraise in other tritrophic systems such as plant-aphid/whitefly/mealybug-parasitoid interactions.

## Results

### PCR analysis of acquisition and persistence of *C*Las in *Tamarixia radiata*

**Fig. 1:**
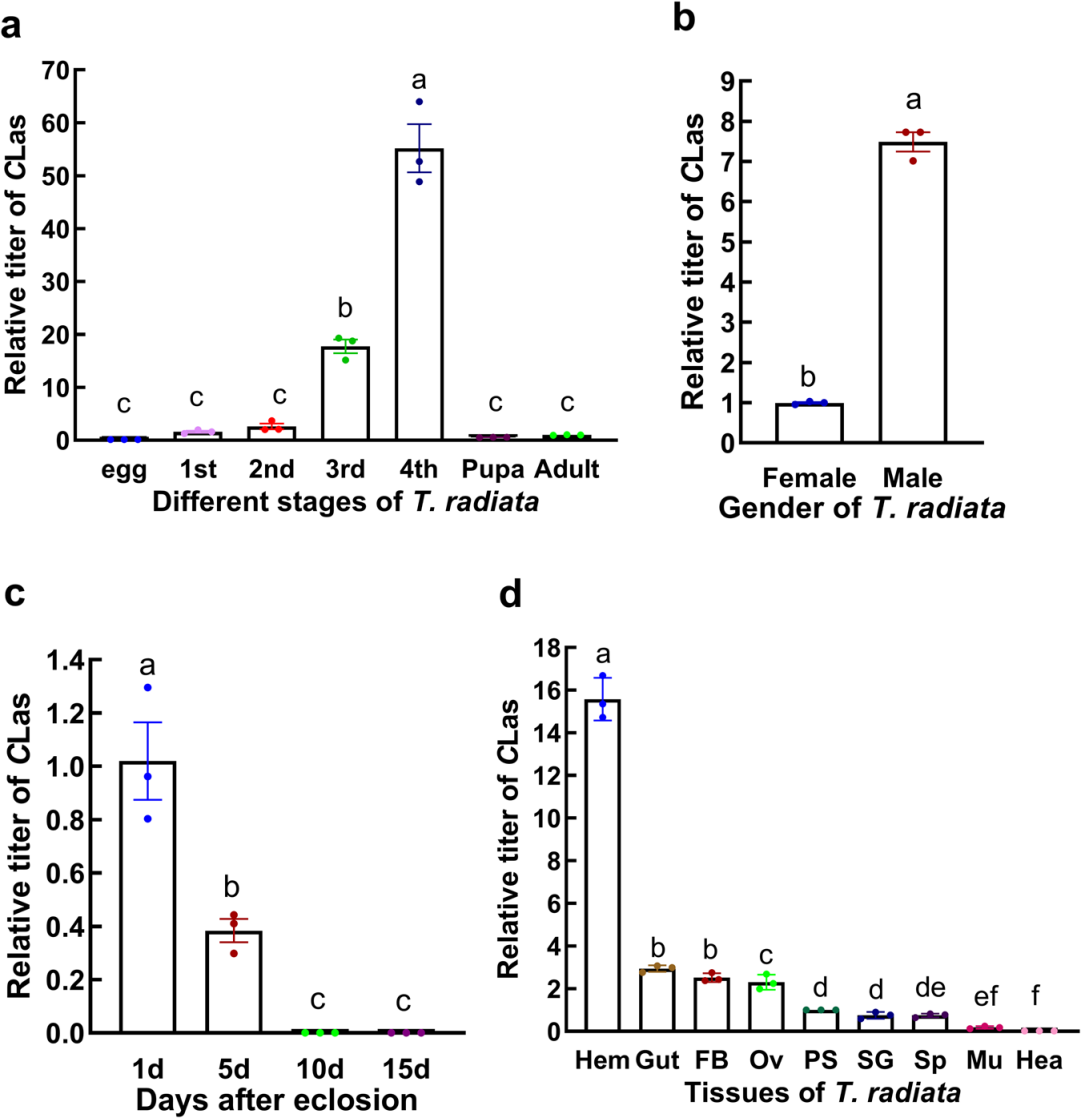
Acquisition and persistence of *C*Las in F_0_*Tamarixia radiata* developed from *C*Las donor ACP hosts. **a** Relative titers of *C*Las in different developmental stages of F_0_ *T. radiata* parasitoids; **b** relative titers of *C*Las in male and female adults of F_0_ *T. radiata*; **c** the retention time of *C*Las in the female adults of F_0_ *T. radiata*; **d** relative titers of *C*Las in different tissue of newly emerged *T. radiata*, Hem: hemolymph, Gut: gut, FB: fat body, Ov: ovary, PS: poison sac, SG: salivary glands, Sp: spermatheca, Mu: chest muscle, Hea: head. The titer of *C*Las was calculated using the method of 2^−ΔΔct^. Error bars represent the mean value ±SE of three replicates Columns with the same letter represent means with no significant difference at *P* < 0.05.

When *T. radiata* eggs hatch inside *C*Las infected psyllids, the *C*Las titers generally increased with the development of *T. radiata* immatures. As expected, *C*Las was not detected in eggs. *C*Las titers in the 3rd and 4th instar larvae were significantly higher than those of 1st or 2nd instar larvae, pupae, and adults (Fig. 1a and Supplementary Data 1). Interestingly, the *C*Las titer of males was significantly higher than that of females (Fig. 1b and Supplementary Data 1). As time went by, *C*Las titers in *T. radiata* female adults decreased gradually to undetectable levels and totally disappeared from qPCR detection on the 10th day after emergence (Fig. 1c and Supplementary Data 1).

Further investigation demonstrated that *C*Las was detectable in different tissues of *T. radiata* adults, but their titers varied among various tissues, for example, the highest titer was in hemolymph, followed by the gut, fat body, ovary, salivary glands, and spermatheca. It was noteworthy that *C*Las was also found in the poison sac and chest muscles of *T. radiata* (Fig. 1d and Supplementary Data 1).

### FISH Localization of *C*Las in different stages of *Tamarixia radiata*

**Fig. 2:**
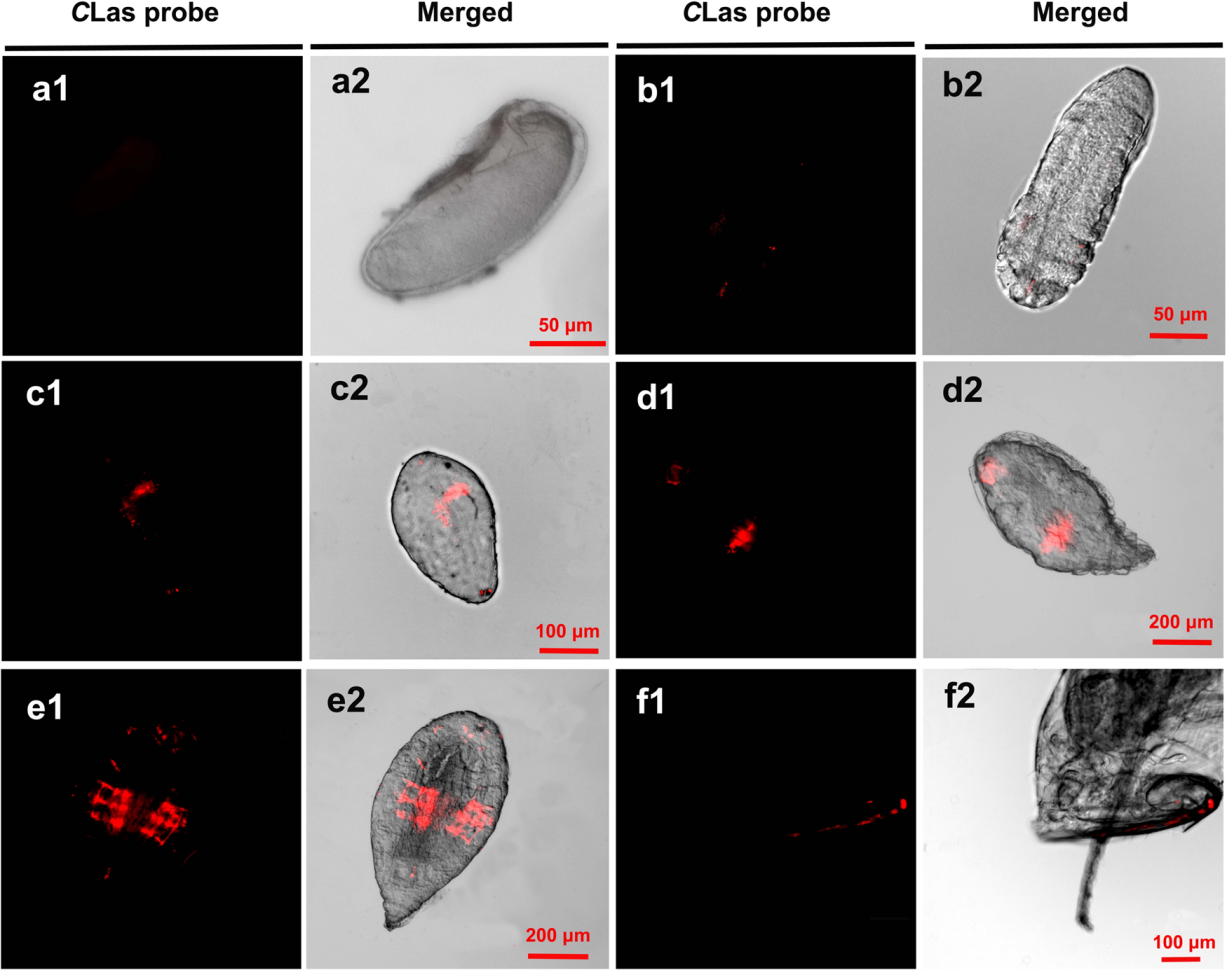
The distribution patterns of *C*Las in F_0_*Tamarixia radiata*. **a1**/**a2**: egg; **b1**/**b2**: 1st instar; **c1**/**c2**: 2nd instar; **d1**/**d2**: 3rd instar; **e1**/**e2**: 4th instar; **f1**/**f2**: the abdominal poisonous sac and ovipositor of female *T. radiata*. Panels **a1**–**f1** show the *C*Las probe (red) in a dark field and **a2**–**f2** show merged images.

*C*Las infections in *T. radiata* were localized visually using fluorescence in situ hybridization. *C*Las fluorescence was visually confirmed in both the poison sac and ovipositor of female *T. radiata* and in all the larval stages of the F_0_ generation, but not in the eggs (Fig. 2a−f), which was consistent with the qPCR detection results (Fig. 1). Over the course of development of *T. radiata* larvae, *C*Las extended its distribution gradually, and as a result, *C*Las had the strongest fluorescent signal in the middle of the abdomen of 4th instar larvae (Fig. 2e). In addition, for the FISH examined 1st to 4th instar larvae of *T. radiata*, 15/20, 17/20, 17/19, and 16/17 were *C*Las infected respectively, and all the infected individuals in each instar stage were consistent to each other with their *C*Las distributions.

### FISH Location of *C*Las in different organs of *Tamarixia radiata*

**Fig. 3:**
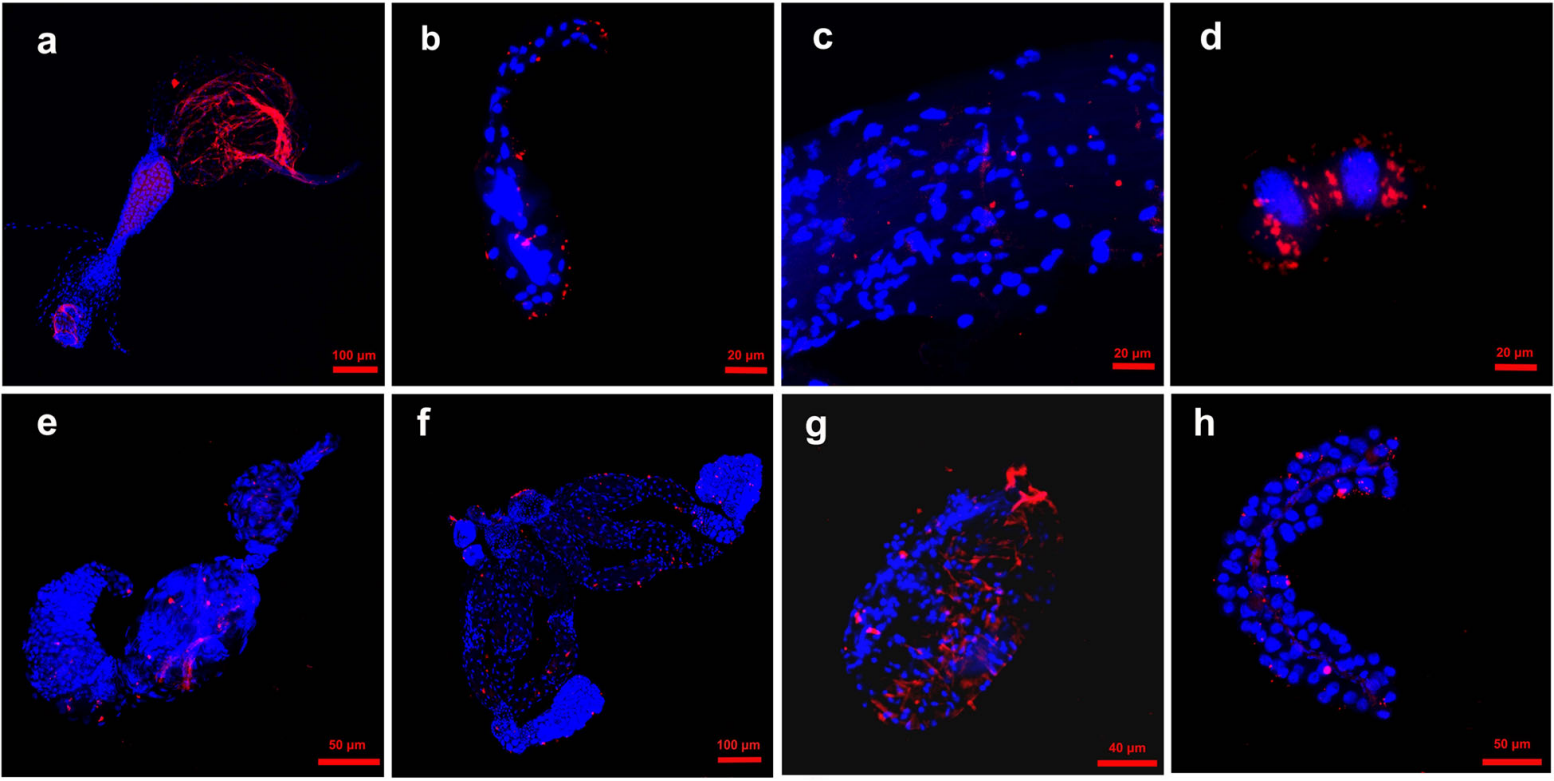
Visualization of *C*Las in different tissues of F_0_*Tamarixia radiata*. **a** gut; **b** salivary glands; **c** chest muscle; **d** fat body; **e** ovary; **f** spermatheca; **g** poison sac; **h** DuFour’s gland. The nuclei were stained with DAPI (blue) and *C*Las were stained with *C*Las probe (red).

*C*Las bacteria could be FISH visualized in the gut, salivary glands, muscle, and fat body (Fig. 3a−d and Supplementary Fig. 1a–d); *C*Las was also localized throughout the tissues of female ovaries and male spermatheca (Fig. 3e, f and Supplementary Fig. 2a, b). In addition, *C*Las had a scattered localization pattern throughout the poison sac (Fig. 3g and Supplementary Fig. 2c) and the DuFour’s gland (Fig. 3h and Supplementary Fig. 2d).

### Maternal transmission of *C*Las between *Tamarixia* generations

**Fig. 4:**
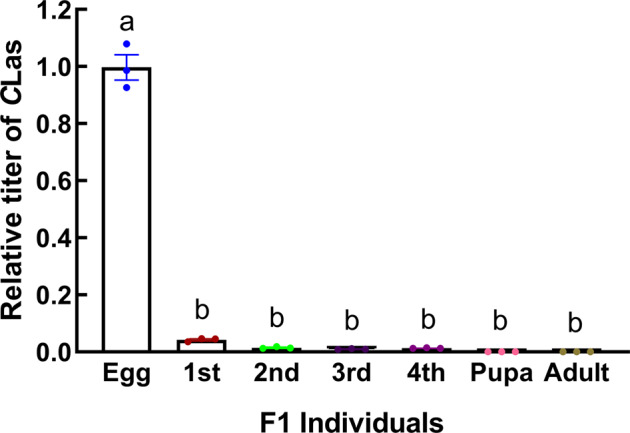
The relative titers of *C*Las in the *Tamarixia radiata* F_1_ generation. The relative titers were calculated using the method of 2^−ΔΔct^. Error bars represent the mean value ±SE. Columns with the same letter represent means with no significant difference at *P* < 0.05. Three replicates of each qPCR were investigated in each column.

The relative titers of *C*Las in different stages of *T. radiata* F_1_ progeny were examined using qPCR. The titer of *C*Las in eggs was significantly higher than that in other life stages; it gradually reduced with larvae development. Eventually, *C*Las was undetectable in the pupal and adult stages of *T. radiata* F_1_ progeny (Fig. 4 and Supplementary Data 1), which indicated that *T. radiata* could not successfully transmit *C*Las vertically to its F_2_ generation.

**Fig. 5:**
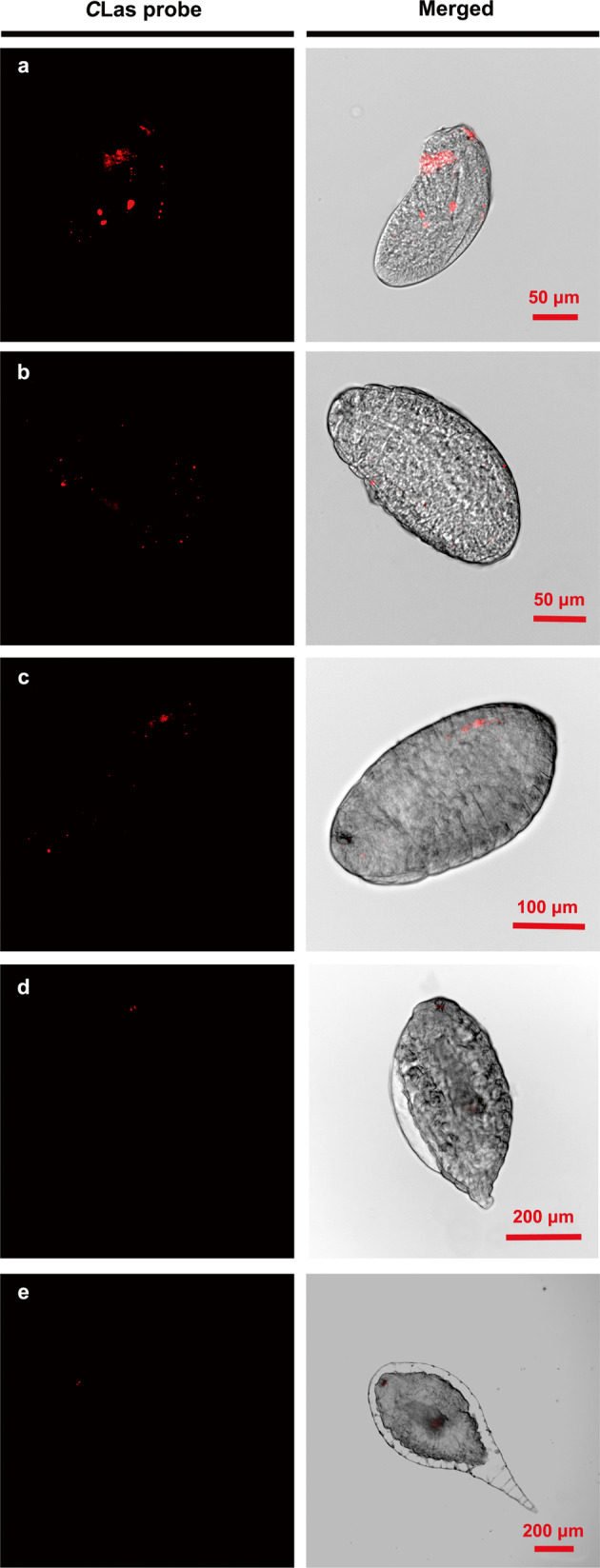
The distribution of *C*Las in the immatures of F_1_*Tamarixia radiata*. **a** egg; **b** 1st instar; **c** 2nd instar; **d** 3rd instar; **e** 4th instar. Panels in the left column show the *C*Las probe (red) in a dark field and in the right column show merged images.

FISH visualization showed strong *C*Las fluorescence signals in F_1_ *T. radiata* eggs exhibiting a scattered distribution pattern (Fig. 5a). With the development of *T. radiata* F_1_ progeny, the fluorescence signal of *C*Las significantly weakened until it almost disappeared in pupal and adult stages (Fig. 5b−e). The FISH visualization results were consistent with the qPCR detection results.

### Localization of *C*Las in ACP following inoculation via parasitoid probes

**Fig. 6:**
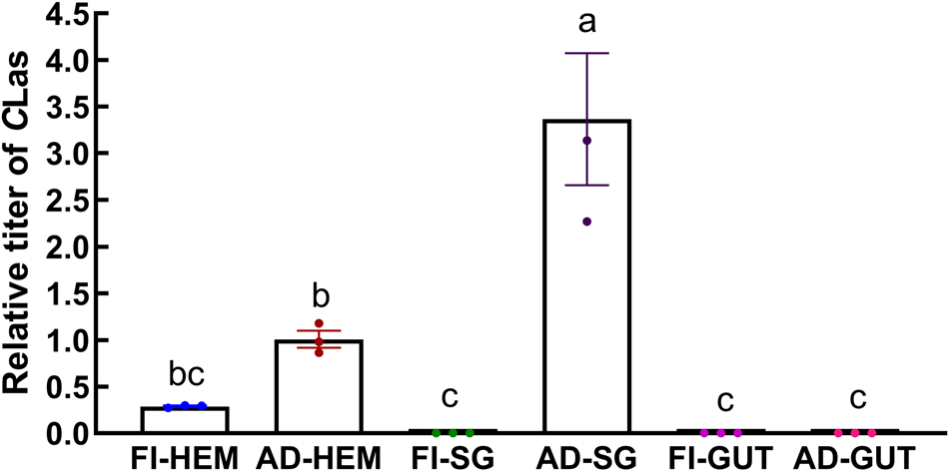
The relative titers of *C*Las in the 5th instar nymphs and adults of *Tamarixia radiata*-inoculated Asian citrus psyllid. FI-HEM: hemolymph of 5th instar nymphs; AD-HEM: hemolymph of adults; FI-SG: salivary glands of 5th instar nymphs; AD-SG: salivary glands of adults; FI-GUT: midgut of 5th instar nymphs; AD-GUT: midgut of adults. The relative titers were calculated using the method of 2^−ΔΔct^ with the house-keeping gene, *β-actin* gene. Error bars represent the mean value ±SE. Columns with the same letter represent means with no significant difference at *P* < 0.05. Three replicates of each qPCR were investigated in each column.

ACP nymphs probed by a *C*Las-infected parasitoid at their 4th instar stage (hereafter referred to as *T. radiata*-inoculated ACP) only had *C*Las detected in their hemolymph, but was not found in the salivary glands or midgut when they developed to 5th instar nymphs (Fig. 6). However, *C*Las was detected both in the hemolymph and salivary glands in 8-day old adults, but still not in their midgut. The *C*Las titer in adults’ hemolymph was significantly higher than that in 5th instar nymphs due to its proliferation. All these findings suggest that after inoculation by *T. radiata*, *C*Las titer accumulates in the hemolymph of 5th instar ACP nymphs, as well as in the hemolymph and salivary glands of 8-day old ACP adults, but fails to enter their midguts (Fig. 6 and Supplementary Data 1).

**Fig. 7:**
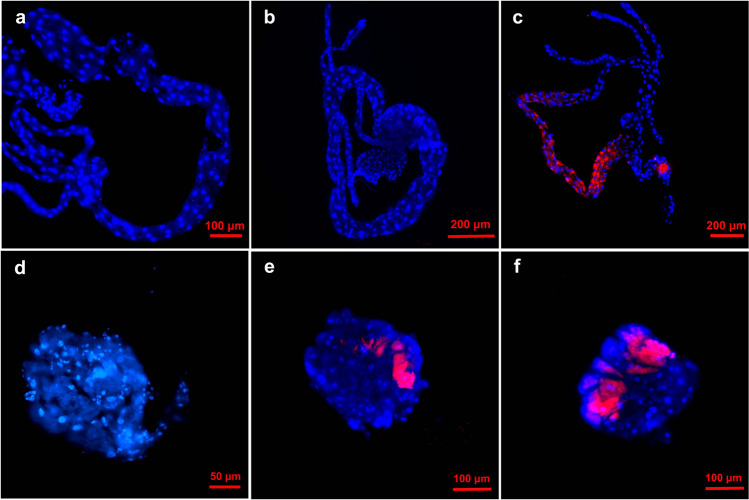
FISH visualization of *C*Las in the midgut and salivary glands of *C*Las-recipient Asian citrus psyllid. **a** midgut of 5th instar ACP nymphs (*C*Las acquired from *T. radiata*); **b** midgut of 8-day old ACP adults (*C*Las acquired from *T. radiata);* **c** midgut of *C*Las positive ACP adults (*C*Las acquired from citrus plants). **d** salivary glands of 5th instar ACP nymphs (*C*Las acquired from *T. radiata*); **e** salivary glands of 8-day old ACP adults (*C*Las acquired from *T. radiata*); **f** salivary glands of *C*Las positive ACP adults (*C*Las acquired from citrus plants). The nuclei were stained with DAPI (blue) and *C*Las were stained with *C*Las probe (red).

FISH was used to confirm the findings of qPCR. In ACP adults that acquired *C*Las directly from citrus plants, *C*Las was clearly visible in midguts (Fig. 7c and Supplementary Fig. 3c); however, no *C*Las FISH signal was found in the midgut of 5th instar nymphs (Fig. 7a and Supplementary Fig. 3a) or 8-day old adults (Fig. 7b and Supplementary Fig. 3b) of the *T. radiata*-inoculated ACP. In addition, *C*Las was not visualized in the salivary glands of these 5th instar ACP nymphs (Fig. 7d and Supplementary Fig. 4a). The *C*Las was visualized in the central area of salivary glands of the 8-day old *T. radiate*-inoculated ACP adults (Fig. 7e and Supplementary Fig. 4b) and when using salivary glands of plant-inoculated ACP adults (Fig. 7 f and Supplementary Fig. 4c). Our findings suggest that *C*Las in *T. radiata*-inoculated ACP accumulates and proliferates during the development of its ACP host. The FISH results were consistent with that of the qPCR results.

### *C*Las transmission to citrus plants by *T. radiata*-inoculated ACP

After 30 days of feeding, *C*Las was not detected in the citrus plants fed on by *T. radiata*-inoculated ACP adults, but was detected in citrus plants fed on by ACP adults that acquired *C*Las from plants (positive control). After 40 days of feeding by *T. radiata*-inoculated ACP, approximately one-third of the citrus leaves were *C*Las positive, compared to 100% of citrus leaves fed on by ACP that acquired *C*Las from plants. However, after 50 days, 100% of the former also tested positive (Supplementary Fig. 5 and Supplementary Data 1). These results demonstrated that *C*Las can proliferate within citrus plants following infection by ACP adults inoculated by *T. radiata* probing.

### Localization of *C*Las in citrus plants fed on by *T. radiata*-inoculated ACP

**Fig. 8:**
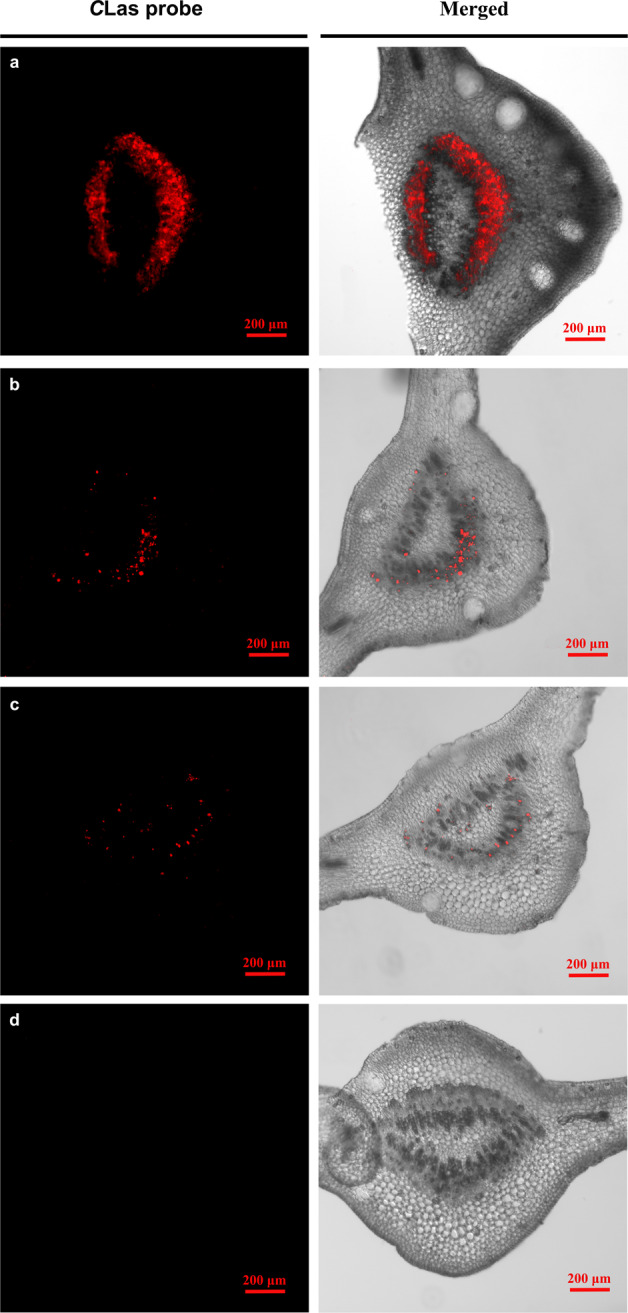
Localization of *C*Las in the recipient citrus plants fed on by *Tamarixia radiata-*inoculated ACP. **a** *C*Las in leaf veins of *C*Las-infected plant; **b** *C*Las in leaf veins that had been fed on by ACP adults that acquired *C*Las from plants; **c** *C*Las in leaf veins that had been fed on by *T. radiata-*inoculated ACP; **d** leaf veins of the plants fed on by healthy ACP.

After plants were fed on by the *T. radiata-*inoculated ACP adults, *C*Las was mainly distributed in the phloem of the citrus leaf veins (Fig. 8a−c). The rankings of fluorescence signals of *C*Las based on visual observations among different citrus leaf samples were: leaf from the *C*Las infected tree (Fig. 8a) >leaf that had been fed on by ACP adults that acquired *C*Las from plants (positive control, Fig. 8b) >leaf that had been fed on by the *T. radiata-*inoculated ACP adults (Fig. 8c). There was no fluorescence signal in the leaf that had been fed on by the *C*Las-free ACP adults (negative control) (Fig. 8d).

### Phylogenetic analysis of *C*Las in different ACP populations and citrus plants

**Fig. 9:**
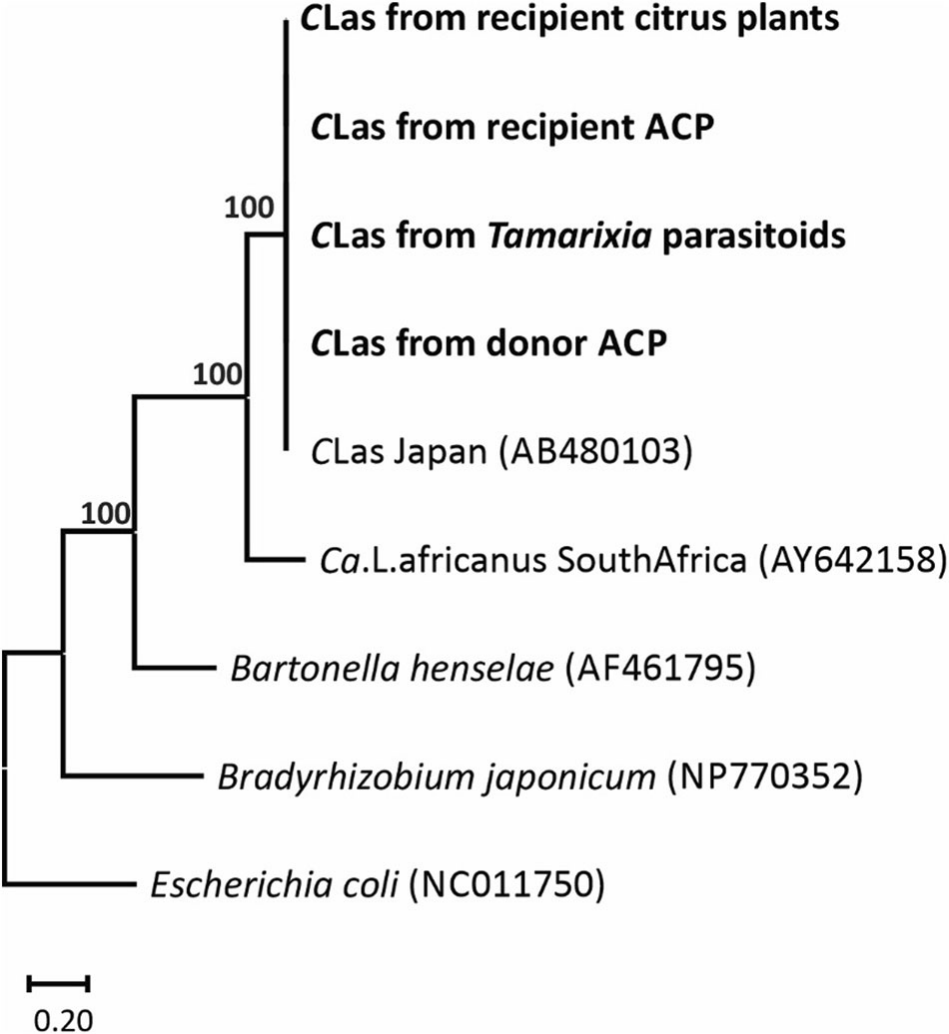
Identification of the *C*Las in different ACP populations and citrus plants. The maximum likelihood phylogenetic tree was generated based on the alignment of *omp* gene sequences of *C*Las and other α-proteobacteria, with 1000 non-parametric bootstrap replications in RAxML. *Escherichia coli* was added as an outgroup.

The *C*Las bacteria had high fidelity during its horizontal transmission from donor ACP to parasitoid, from parasitoid to *T. radiata-*inoculated ACP and from *T. radiata-inoculated* ACP to recipient citrus plant. There was no variation in their *omp* gene sequences (GenBank accession numbers: MT191373-MT191376). Based on their *omp* gene sequences, all of the *C*Las isolates grouped into one branch belonging to the Asia *Candidatus* Liberibacter asiaticus (Fig. 9).

## Discussion

The current study demonstrates, for the first time to the best of our knowledge, that the parasitoid *T. radiata* can act as a vector of *C*Las. It has been proven that *C*Las pathogen can localize in different tissues and organs of *T. radiata*, after it is initially picked up during its development in *C*Las-infected ACP hosts. The *C*Las could be detected by qPCR for at least 5 days in *T. radiata* adult females following their emergence from *C*Las-infected ACP nymphs. This is similar to findings in a previous study by Ahmed et al.^15^ that the bacterial endosymbiont, *Wolbachia*, could be detected in the whitefly parasitoid adult female up to at least 5 days after they had fed on, or probe checked, *Bemisia tabaci* nymphs that were infected with *Wolbachia*.

Before this study, parasitoids, and mites had been found to transmit bacterial endosymbionts horizontally in some previous studies. For example, Jaenike et al.^16^ found that the ectoparasitic mites, *Macrocheles subbadius*, feeding on insect hemolymph, can pick up the bacterial endosymbiont *Spiroplasma* species from infected *Drosophila nebulosa* females and horizontally transmit it to *Drosophila willistoni*. Progeny of the recipient *D. willistoni* were infected, indicating successful maternal transmission of *Spiroplasma* into a new host species. Gehrer and Vorburger^17^ revealed that the parasitoid *Aphidius colemani* can transmit the bacterial endosymbiont *Hamiltonella defensa* by sequentially stabbing infected and uninfected individuals of their aphid hosts *Aphis fabae*, thus establishing new, heritable infective lines. In addition, our previous study^15^ reported that the parasitoid *Eretmocerus* sp. nr. *furuhashii* can phoretically acquire the bacterial endosymbiont *Wolbachia* when probe checking infected whitefly hosts. They can then transmit it to uninfected whitefly hosts horizontally. All previous parasitoid-based horizontal transmissions of bacteria were investigated in endosymbionts only. Our study has revealed, for the first time to the best of our knowledge, the unexpected role of a *Tamarixia* parasitoid in transmitting a plant bacterial pathogen and raises concern about the potential for adverse effects on ACP biological control as it relates to limiting the spread of HLB to uninfected citrus. In addition, Ahmed et al.^15^ revealed that both the probing and feeding of *Eretmocerus* sp. nr. *furuhashii* can result in the horizontal transmission of *Wolbachia* between whitefly hosts, whereas only the female ACP nymphs that were probed by *T. radiata* and survived were investigated in the current study. If the male and female ACP nymphs that survived *T. radiata* probing are also taken into consideration, the unexpected risk of *T. radiata* vectoring *C*Las transmission may increase, since we have also shown that the *C*Las titer in male *T. radiata* was higher than even in females.

In other studies, its been shown that endosymbiotic bacteria transmit horizontally with poor fidelity^18–20^ or fail to persist^21^ between different species. Our previous study revealed *Rickettsia* kept 100% fidelity when it was transmitted horizontally between the whitefly *Bemisia tabaci* MEAM1 and MED populations via cotton plants^22^. In agreement with the previous findings^22^, the horizontal transmission of *C*Las vectored by *T. radiata* in the current study showed 100% fidelity between the *C*Las bacteria in donor ACP, *T. radiata* parasitoids, *T. radiata*-inoculated ACP, and recipient citrus plants.

It is commonly believed that the alimentary canal and salivary glands are the key barriers for the transmission and spread of bacterial pathogens from insect vectors to plants^23–26^. Bacteria usually need to penetrate and accumulate in specific cells of the salivary glands before being successfully transmitted into plants through the salivary glands of insect hosts^27^. Wu et al.^28^ reported that *C*Las multiplication was detected in the hemolymph and salivary glands of ACP adults when the bacterium was acquired by ACP nymphs. In conjunction with the previous study, our current study also revealed that *C*Las could multiply in the hemolymph and salivary glands of the ACP adults that developed from nymphs which were probed by *C*Las-vectored *T. radiata* parasitoids. The presence of *C*Las in the citrus leaves fed on by *T. radiata-*inoculated ACP suggests that the parasitoid *T. radiata* can be a potential vector, transmitting *C*Las from the infected to the healthy citrus plants through acquisition from *C*Las infected ACP and then transmission to uninfected ACP that then feed on citrus plants.

When releasing *T. radiata* parasitoids to control ACP in orchards, the *C*Las infection status of ACP and the citrus plants should be determined. This is because *T. radiata* parasitoids, which have fed upon or parasitized the *C*Las-infected ACP, can transmit *C*Las to other ACP at new locations and therefore introduce *C*Las to healthy plants in the orchard. Even if *T. radiata* parasitoids are reared on *C*Las-free ACP and *C*Las-free plants, then released into the orchard, where there are *C*Las-infected ACP and trees that are either *C*Las-free or asymptomatic with undetectable or unknown *C*Las titer, *C*Las can still be picked up and transmitted. Since *C*Las can spread throughout an entire orchard within a year before any disease symptoms appear, according to the study of Lee et al.^29^, if there are a few *C*Las-infected asymptomatic trees in the orchard, or if a few *C*Las-vectored *T. radiata* parasitoid adults accidentally get introduced into a *C*Las*-*free orchard, then there might be a chance that *C*Las invasion could be initiated. It should be noted that our current experiments were performed under laboratory conditions and may not be representative of the full suite of biotic and abiotic factors present in field conditions. Our main study revealed that parasitoid vector *T. radiata* can contract and potentially transmit plant pathogen *C*Las, which will potentially diminishing the benefits it confers as biological control agent. Future research should be conducted using agent*-*based models to determine the impact of this new mode of transmission in HLB spread and to evaluate the potential risk of using *T. radiata* as a biological agent against ACP in citrus orchards.

**Fig. 10:**
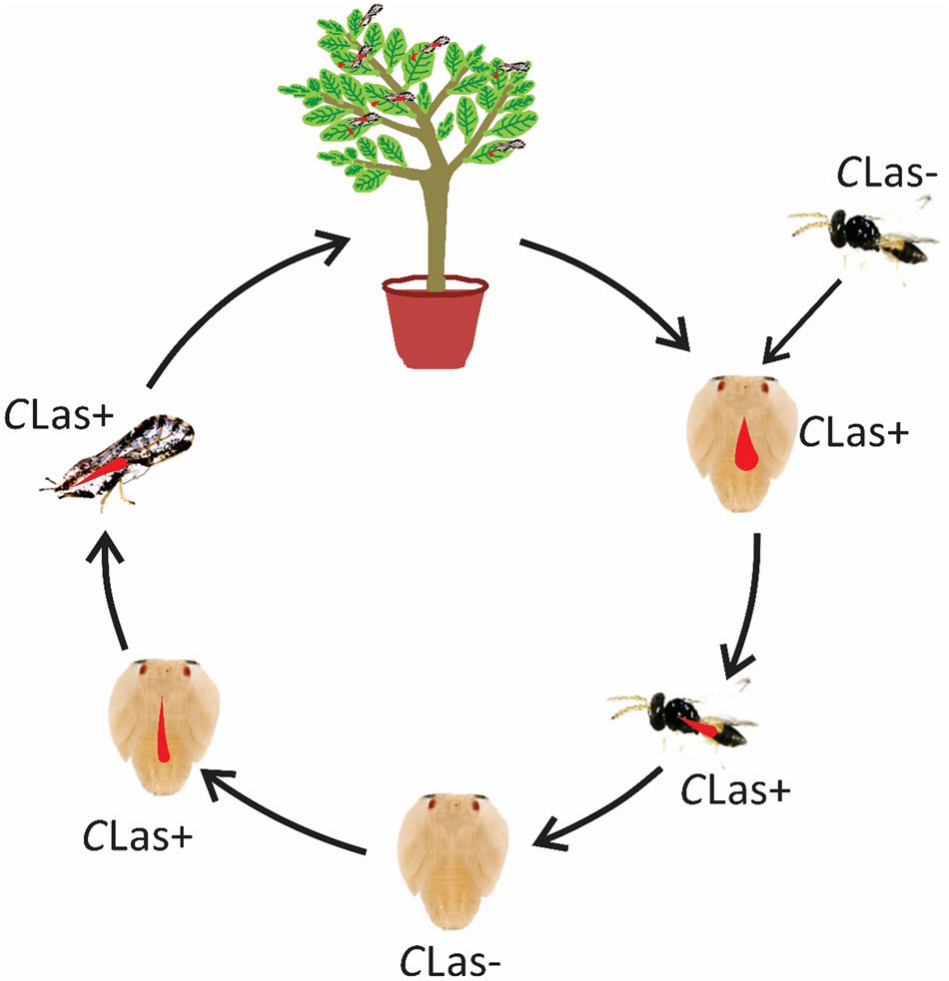
Schematic overview of *C*Las transmission vectored by *Tamarixia radiata* parasitoid. When ACP nymphs feeding on the *C*Las positive citrus plants, its parasitoid *Tamarixia radiata* females can acquire *C*Las from these *C*Las-infected ACP nymphs and carry it in their ovipositors. After probe checking *C*Las negative ACP hosts with ovipositor, *Tamarixia*can transinfect *C*Las into the hemolymph of probed ACP. If parasitized ACP nymphs survive from the parasitizing check, *C*Las can spread into the salivary glands of the ACP host after proliferation. When the *C*Las-recipient ACP individuals develop into adults and move to healthy citrus plants, *C*Las can be transmitted to citrus leaves during recipient ACP feeding, completing the transmission circle of the pathogen from *C*Las-infected citrus to ACP, to parasitoid, from parasitoid to *C*Las uninfected-ACP and from *C*Las-recipient ACP to-citrus plant.

In conclusion, our current study reveals that *C*Las can be picked up by the parasitoid *T. radiata* during their development in *C*Las-infected ACP nymphs, and that it can persist in the parasitoid adult for at least 5 days following wasp emergence. During its persistence in the parasitoid, this bacterium can be transmitted to a new ACP host by the wasp feeding or probe checking for oviposition. Eventually, the *Tamarixia*-inoculated ACP can transmit *C*Las to healthy citrus plants during their feeding (Fig. 10). To our knowledge, this is the first report of an insect natural enemy, actively applied for the biological control of a given pest, could potentially contribute to transmission of a plant pathogen and therefore spread of the resulting plant disease.

## Materials and methods

### Insect rearing

A *C*Las negative colony of ACP was initially collected from *C*Las-free *Murraya exotica* L. growing in the ornamental landscape of South China Agricultural University (SCAU, Guangzhou, China) in May 2014. Then it was reared on potted *M. exotica* in a greenhouse at SCAU. *M. exotica* plants were pruned regularly to promote the growth flushes necessary to stimulate ACP oviposition. The ACP populations were periodically (at least once a month) tested to ensure the colony was *C*Las-free using nested quantitative PCR detection according to the method described by Coy et al.^30^.

The parasitoid *T. radiata* used in the current study was initially collected from ACP hosts on *M. exotica* plants in the above-mentioned location during June 2015. Its population was maintained in rearing cages (60 × 60 × 60 cm) using a *C*Las-free ACP-*M. exotica* rearing system under laboratory conditions (26 ± 1 °C, RH 80 ± 10% with L:D = 14:10 photoperiods in insect incubators).

### Host plants

*C*Las-free and *C*Las-infected plants of *Citrus reticulata* Blanco cv. Shatangju were used in the current study. Both plant types were obtained from The Citrus Research Institute of Zhaoqing University (Guangdong, China). The *C*Las-infected plants were inoculated by shoot grafting. All plants were approximately 4-year old and 1.2−1.5 m in height, separated in nylon net greenhouses (70 mesh per inch2) at two different locations about 2.2 km apart in SCAU. Again, nested qPCR detection was performed periodically (at least once a month) to test for the presence or absence of *C*Las in the citrus plants according to the method described by Coy et al.^30^.

### Acquisition and persistence of *C*Las in *Tamarixia radiata*

When new shoots of *C*Las-infected *C. reticulata* plants were grown to 5–8 cm, 20 pairs of 1 week-old ACP adults were introduced into one nylon bag covering one fresh shoot to lay eggs for 48 h. When the progeny of ACP developed through to 4th or 5th instar nymph (*C*Las-donor ACP), which are the stages preferred by *T. radiata* parasitoids, 150 of the ACP nymphs were randomly selected and the remaining ones were removed. Following this, 10 pairs of 3-day old *T. radiata* adults, randomly selected from the population that has been tested to be *C*Las-free, were introduced into the nylon bag in order to parasitize the 4th or 5th instar ACP nymphs for 48 h before being recaptured. Then the potentially parasitized ACP nymphs together with the citrus plants were cultured in a plant growth chamber (Jiangnan Instrument Company, RXZ-500D, at 26 ± 1 °C, 60 ± 2% RH and 14:10 h L:D photoperiod of 3,000 lx illumination).

When the progeny of *T. radiata* (considered F_0_ generation) developed to 3-day egg, 1st to 4th instar larvae, pupae, and adult stages respectively, they were identified and collected with the assistance of a stereomicroscope. DNA of each stage sample was extracted using the TIANamp Genomic DNA Kit (TIANGEN, Beijing, China) for *C*Las qPCR detection and titer quantification. Thirty eggs, 20 individuals of 1st or 2nd instar, 10 individuals of 3rd or 4th instar larvae or pupa, as well as three individuals of female or male adults were subsequently ground together to represent each life stage in qPCR, and each stage qPCR detection was repeated three times.

The primers used for *C*Las qPCR detection were LJ900 primers, (F5′-GCCGTTTTAAC ACAAAAGATGAATATC-3′, R5′-ATAAATCAATTTGTTCTAGTTTAC GAC-3′), and 18S rRNA gene of *T. radiata* (F5′-AAACGGCTACCACATCCA-3′, R5′-ACCAGACT TGCCCTC CA-3′)^31^ was used as an internal control for DNA normalization and quantification. In order to normalize the qPCR values, each qPCR reaction was performed in three independent runs using SYBR Premix Ex Taq (Takara, Dalian, China) in Bio-Rad CFX Connect™ Real-Time PCR Detection System, with a protocol of initial denaturation at 95 °C for 3 min, followed by 40 cycles at 95 °C for 10 s, 60 °C for 20 s and 72 °C for 30 s.

To monitor the *C*Las persistence in *T. radiata*, newly emerged female adults of *T. radiata* (considered F_1_ generation) were collected from the above experiment and fed with 20% honey water. After 1, 5, 10, and 15 days, 10 parasitoids were recaptured, subsequently ground for DNA extraction and *C*Las titer detection and quantification using qPCR. The protocol of DNA extraction and qPCR reaction was the same as above, and qPCR quantification was repeated three times for each treatment.

### Localization patterns of *C*Las in *Tamarixia radiata*

#### Localization patterns of CLas in different instars of T. radiata

Fluorescent in situ hybridization (FISH) was used to visualize the distribution of *C*Las in *T. radiata* exposed to *C*Las positive ACP, following the method of Gottlieb et al.^32^ with a slight modification. Eggs and different larval instars of *T. radiata* were collected and fixed in Carnoy’s solution (chloroform-ethanol-glacial acetic acid [6:3:1,vol/vol] formamide) overnight at 4 °C. After fixation, the samples were washed three times in 50% ethanol with 1× phosphate buffered saline (PBS) for 5 min. Then the samples were decolorized in 6% H_2_O_2_ in ethanol for 12 h, after which they were hybridized overnight in 1 ml hybridization buffer (20 mM Tris-HCl pH 8.0, 0.9 M NaCl, 0.01% sodium dodecyl sulfate, 30% formamide) containing 10 pmol of fluorescent probes/ml in a 37 °C water bath under dark conditions. The *C*Las probe used for FISH was 5′-Cy3-GCCTCGCGACTTCGCAACCCAT-3′. Finally, the stained *T. radiata* samples were washed three times in a washing buffer (0.3 M NaCl, 0.03 M sodium citrate, 0.01% sodium dodecyl sulfate, 10 min per time). After the samples were whole mounted and stained, the slides were observed and photographed using a Nikon eclipse Ti-U inverted microscope. For each stage sample, approximately 20 individuals were examined to confirm the results.

#### Localization patterns of CLas in different organs of T. radiata

Different organs (gut, fat body, ovary, poison sac, salivary glands, spermatheca, and chest muscle) were dissected from newly emerged adults of *T. radiata* in 1× phosphate buffered saline (PBS) under a stereomicroscope using a depression microscope slide and a fine anatomical needle. After a sufficient number of each tissue sample was collected (20 or more), the tissues were washed three times with 1 × PBS, followed by the fixation, decolorization, and hybridization procedures as outlined above, except that this time of decolorization was 2 h. After hybridization, nuclei in the different organs were counterstained with DAPI (0.1 mg/ml in 1 × PBS) for 10 min, then the samples were transferred to slides, mounted whole in hybridization buffer, and viewed using confocal microscopy (Nikon, Japan).

### Maternal transmission of *C*Las between *Tamarixia* generations

Five groups of experiments were used to clarify whether *C*Las can be transmitted vertically between different *T. radiata* generations. In the first group, 60 pairs of newly emerged *T. radiata* adults from the *C*Las-infected ACP colony (potential *C*Las-acquired parasitoid adults, F_0_ generation) were introduced into 60 nylon bags (one female per cage). Each bag covered one fresh citrus plant shoot with one marked *C*Las-free 4th instar nymph of ACP, the parasitoid females were given 24 h to oviposit, then transferred to another four groups successively to oviposit with intervals of 24 h before they were recaptured for *C*Las-PCR detection (58/60 and 56/60 *T. radiata* females and males respectively were *C*Las-infected). Only the progeny (F_1_ generation) in which parasitoid parents were both *C*Las-infected continued to be investigated.

When the F_1_ progeny of *C*Las-infected parasitoid females developed to egg, larval, pupal, and adult stages respectively, they were collected and divided into two groups; in one group samples were used for the qPCR detection of the *C*Las titer, and the other group was used for the FISH visualization of *C*Las. The qPCR and FISH analysis protocols of *C*Las as well as the number of tested individuals were the same as previously outlined. Each stage was repeated three times.

### *C*Las detection in *T. radiata*-inoculated ACP

#### Quantitative PCR detection of CLas

Approximately 60 newly emerged parasitoid adult females from *C*Las-infected ACP hosts (potential *C*Las-acquired parasitoid adults) were collected using an aspirator. They were first starved for 5 h, then released into finger tubes (diameter 6 mm × length 30 mm); one female per tube containing one 4th instar nymph of *C*Las-free ACP (this was treated as one experimental replicate). The probing behavior of the parasitoids was observed under a stereomicroscope, after which the parasitoids were recaptured for *C*Las PCR detection (similar to the above experiment, approximately 95% were *C*Las-infected). Only those 4th instar ACP nymphs, probed for egg-laying by a *C*Las-infected parasitoid but survived from the probing (the averaged proportion of such samples was 5.36 ± 0.47% and were 100% *C*Las infected), were transferred onto fresh *C*Las-free M. exotica shoots to complete their development (hereafter referred as “*T. radiata*-inoculated ACP”). The experiment was repeated in 32 parallel replicates (Supplementary Table 1), in which 103 *T. radiata*-inoculated ACP nymphs were finally obtained.

Following the above, thirty *T. radiata-*inoculated ACP nymphs were collected when they developed into 5th instar nymphs (the stage when infection proliferation might have just begun since the infection was introduced at the 4th instar). In addition, thirty 8-day old adults that developed from the *T. radiata-*inoculated ACP nymphs were also collected. This was because the results in Wu et al.^28^ revealed that the proportion of *C*Las-infected ACP individuals exceeds 90% at the 12th day after infection acquisition, while ACP takes 4 days to develop into an adult from 5th instar stage. Their alimentary canals and salivary glands were dissected under a stereomicroscope using the methods of Ammar et al.^33^, and hemolymphs were collected with a 10 μl pipette tip using the method of Killiny et al.^34^. The DNA of the alimentary canals, salivary glands and hemolymphs were extracted using TIANamp Micro DNA Kit (Tiangen, Beijing, China), and the relative titers of *C*Las in each tissue of ACP nymphs and adults were detected by qPCR with of LJ900. The *β-actin* gene of ACP (F 5′-CCCTGGACTTTGAACAGGAA-3′; R 5′-CTCGTGGATACCGCAAGATT-3′) was selected as an internal control for data normalization and quantification^35^. For each sample, qPCR detection was repeated three times.

#### FISH visualization of CLas

The alimentary canals and salivary glands of 5th instar nymphs and 8-day old adults of *T. radiata-*inoculated ACP were dissected as described above, and the distribution of *C*Las was visualized by FISH and confocal microscopy. The alimentary canals and salivary glands of *C*Las-infected ACP nymphs and adults (collected from *C*Las-infected citrus plants) were used as a positive control, and five to ten samples were detected by FISH for each tissue.

#### *C*Las transmission from *T. radiata-*inoculated ACP to citrus plants

According to the above experimental results, if the *C*Las could be detected in the salivary glands of the 8-day old ACP adults (*T. radiata-*inoculated ACP), 30 more of these adults were randomly selected to inoculate on fresh shoots of *C*Las-free citrus. ACP adults that acquired *C*Las from plants and *C*Las-free ACP adults were used as positive and negative controls respectively.

After 20, 30, 40, and 50 days of feeding samples of the citrus leaves fed on by *T. radiata-*inoculated ACP (named as *C*Las-recipient citrus leaves), fed on by ACP that acquired *C*Las from plants (positive control), and fed on by *C*Las-free ACP (negative control) were cut (1 cm2). Their DNAs were extracted using DNAsecure Plant Kit (Tiangen, Beijing, China). The infections of *C*Las in these plants were detected by nested PCR based on the methods of Jagoueix et al.^36^ and Deng et al.^37^. The experiment was repeated in six plants for each of 20, 30, 40, and 50 days feeding duration, and the infection rates of *C*Las were calculated.

### Localization of *C*Las in citrus plants fed on by *T. radiata-*inoculated ACP

In order to further confirm the infection of *C*Las in the recipient citrus leaves, FISH was used to visualize the localization of *C*Las. According to the above experimental results, after being fed on for 50 days by the *T. radiata-*inoculated ACP adults, citrus leaf sections containing the midrib were cross-sliced in 30 µ sections using a cryostat (CM1950, Leica, Germany). The leaf samples were prepared for FISH vitalization according to the protocol described by Gottlieb et al.^32^. Citrus leaves from the plant that had been fed on by ACP adults that acquired *C*Las from plants and *C*Las-free ACP adults were used as positive and negative controls, respectively. Five to 10 leaf samples were detected by FISH for each treatment.

### Phylogenetic analysis of *C*Las bacteria in different ACP populations and citrus plants

To assess the identity of the *C*Las bacteria in *C*Las donor ACP, *C*Las vectored parasitoids, *T. radiata-*inoculated ACP and recipient citrus leaves, the outer membrane protein gene (*omp*) of *C*Las was PCR amplified with the primers HP1asinv (5′-GATGATAGG TGCATAAAAGTACAGAAG-3′) and Lp1c (5′-AATACCCTTATGGGATACAAAAA-3′) following the procedure described in Bastianel et al.^38^. Then the PCR products were sent for sequencing after visualizing the expected bands on 1% agarose gels.

All the DNA sequences of *C*Las *omp* gene were edited and aligned manually using Clustal X1.83^39^ in Mega 6^40^. The best model and partitioning scheme were chosen using the Bayesian information criterion in PartitionFinder v.1.0.1^41^. Phylogenetic analysis was undertaken using a maximum likelihood (ML) method with 1000 non-parametric bootstrap replications in RAxML^42^. *Escherichia coli* was used as an outgroup.

### Statistics and reproducibility

Taking *18S rRNA* gene of *T. radiata* and the *β-actin* gene of ACP as housekeeping genes, the relative titers of *C*Las in different stages and different tissues of *T. radiata* and ACP were calculated using the method of 2^[−ΔΔct 43^. For the parallel experiments that had more than three replicates the differences were compared using analysis of variance (ANOVA) with SPSS 18.0 at a significance level α = 0.05; while for *C*Las titer, two-sample comparison between genders of *Tamarixia* adults analysis was performed using paired t-test. Fluorescent pictures were processed using Photoshop CS5 software.

### Reporting summary

Further information on research design is available in the Nature Research Reporting Summary linked to this article.

## Supplementary information

Peer Review File

Supplementary Information

Description of Additional Supplementary Files

Supplementary Data 1

Reporting Summary

## Data Availability

The *omp* gene sequences of *C*Las in the donor ACP, *Tamarixia radiata* parasitoids, *T. radiata*-inoculated ACP, and recipient citrus plants are available in GenBank (accession numbers: MT191373–MT191376); Correspondence and requests for materials should be addressed to Bao-Li Qiu.
