# Supplementary material for: Parasitoid vectors a plant pathogen, potentially diminishing the benefits it confers as a biological control agent
Source: Commun Biol. 2021 Nov 25;4:1331. doi: 10.1038/s42003-021-02851-2 (PMC8617049; doi:10.1038/s42003-021-02851-2)
Supplement: Supplementary file 2 — Supplementary Information [file 42003_2021_2851_MOESM2_ESM.pdf]

Supplementary online files

Descriptions of additional supplementary files

Supplementary Table 1 Survival rate of *Tamarixia radiata*-inoculated ACP.

Supplementary Figure 1 Visualization of *C*Las in different tissues of *Tamarixia radiata*.

Supplementary Figure 2 Visualization of *C*Las in the reproductive organs and venomous organs of *Tamarixia radiata*.

Supplementary Figure 3 FISH visualization of *C*Las in the midgut of *C*Las-recipient Asian citrus psyllid.

Supplementary Figure 4 FISH visualization of *C*Las in salivary glands of *C*Las-recipient Asian citrus psyllid.

Supplementary Figure 5 *C*Las transmission efficiency from *C*Las-recipient ACP to citrus plants.

Supplementary Table 1 Survival rate of *Tamarixia radiata*-inoculated ACP.

Number of repetitions	Number of ACP	Number of survivors	Survival rate (%)
Replicate 1	60	3	5.00
Replicate 2	60	5	8.33
Replicate 3	60	2	3.33
Replicate 4	60	2	3.33
Replicate 5	60	3	5.00
Replicate 6	60	3	5.00
Replicate 7	60	2	3.33
Replicate 8	60	6	10.00
Replicate 9	60	3	5.00
Replicate 10	60	4	6.67
Replicate 11	60	1	1.67
Replicate 12	60	2	3.33
Replicate 13	60	4	6.67
Replicate 14	60	6	10.00
Replicate 15	60	5	8.33
Replicate 16	60	2	3.33
Replicate 17	60	3	5.00
Replicate 18	60	1	1.67
Replicate 19	60	5	8.33
Replicate 20	60	1	1.67
Replicate 21	60	4	6.67
Replicate 22	60	5	8.33
Replicate 23	60	3	5.00
Replicate 24	60	2	3.33
Replicate 25	60	6	10.00
Replicate 26	60	2	3.33
Replicate 27	60	3	5.00
Replicate 28	60	2	3.33
Replicate 29	60	1	1.67
Replicate 30	60	5	8.33
Replicate 31	60	2	3.33
Replicate 32	60	5	8.33

The averaged proportion of such samples was 5.36±0.47%

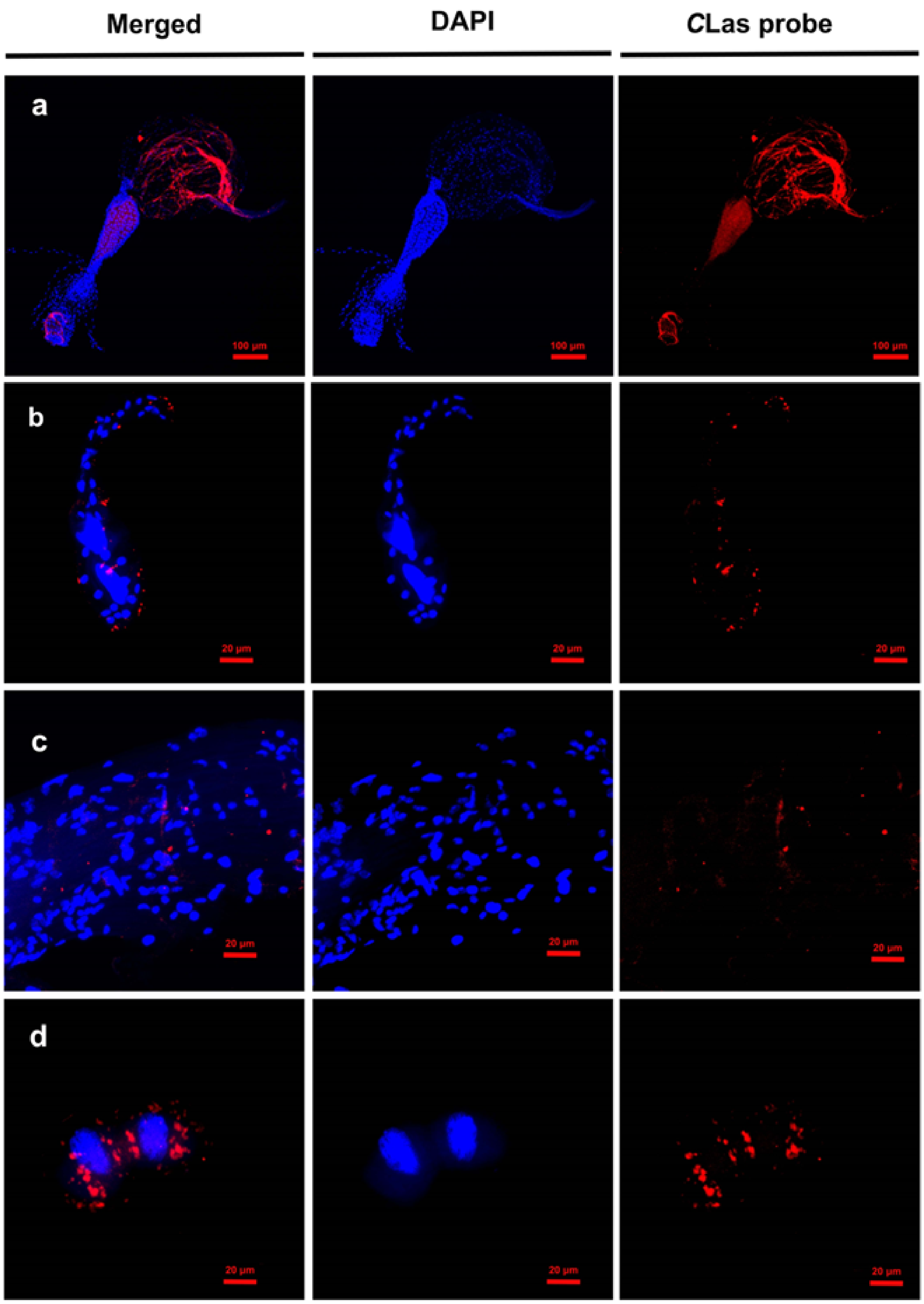

Supplementary Figure 1. Visualization of *C*Las in different tissues of *Tamarixia radiata*.

a, gut; b, salivary glands; c, muscle; d, fat body. The left column shows merged images, the middle column shows the DAPI staining (blue) and the right column shows the *C*Las probe (red) images.

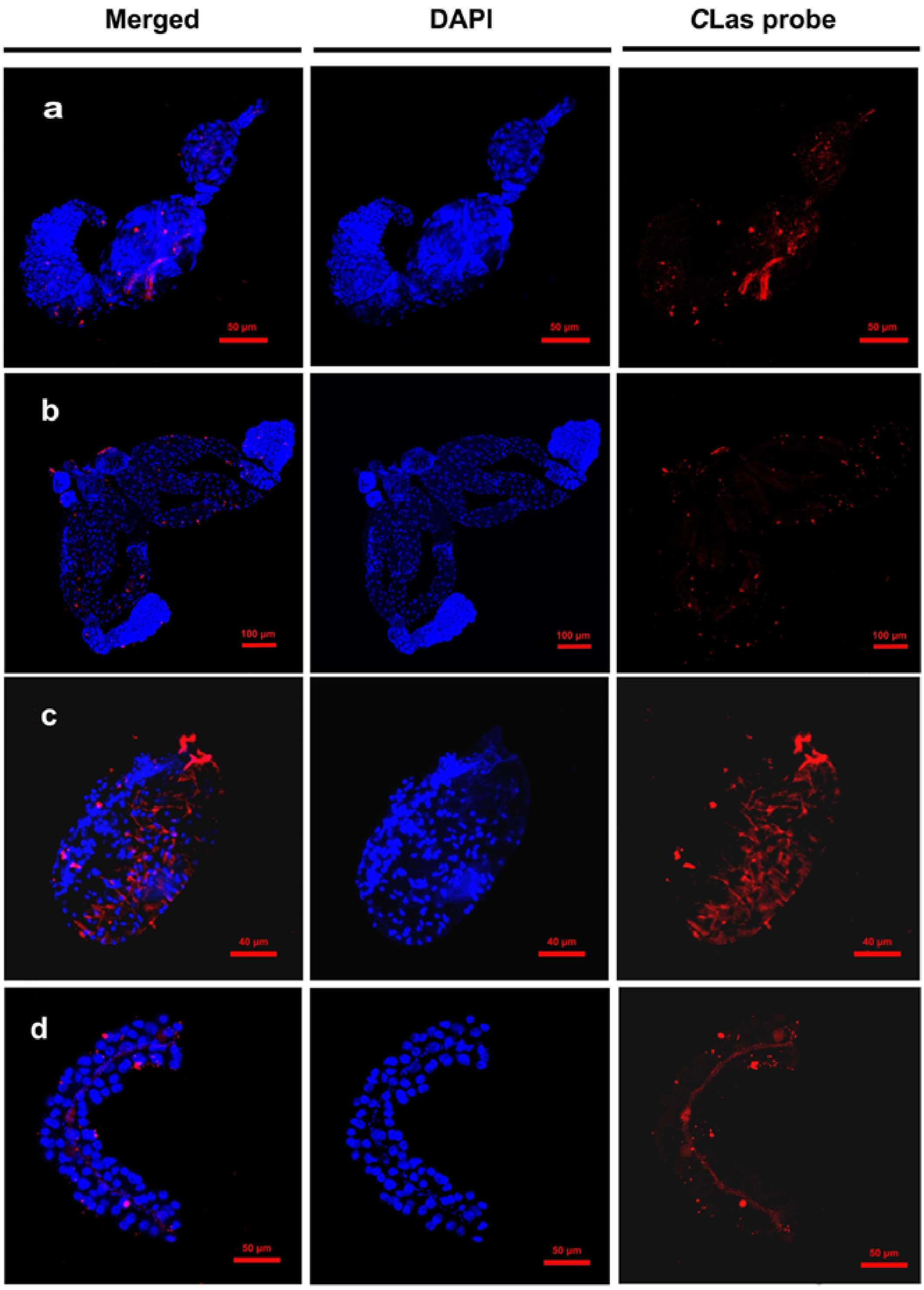

Supplementary Figure 2. Visualization of *C*Las in the reproductive organs and venomous organs of *Tamarixia radiata*. a, ovary; b, spermatheca; c, poison sac; d, DuFour's gland. The left column shows merged images, the middle column shows the DAPI staining (blue) and the right column shows the *C*Las probe (red) images.

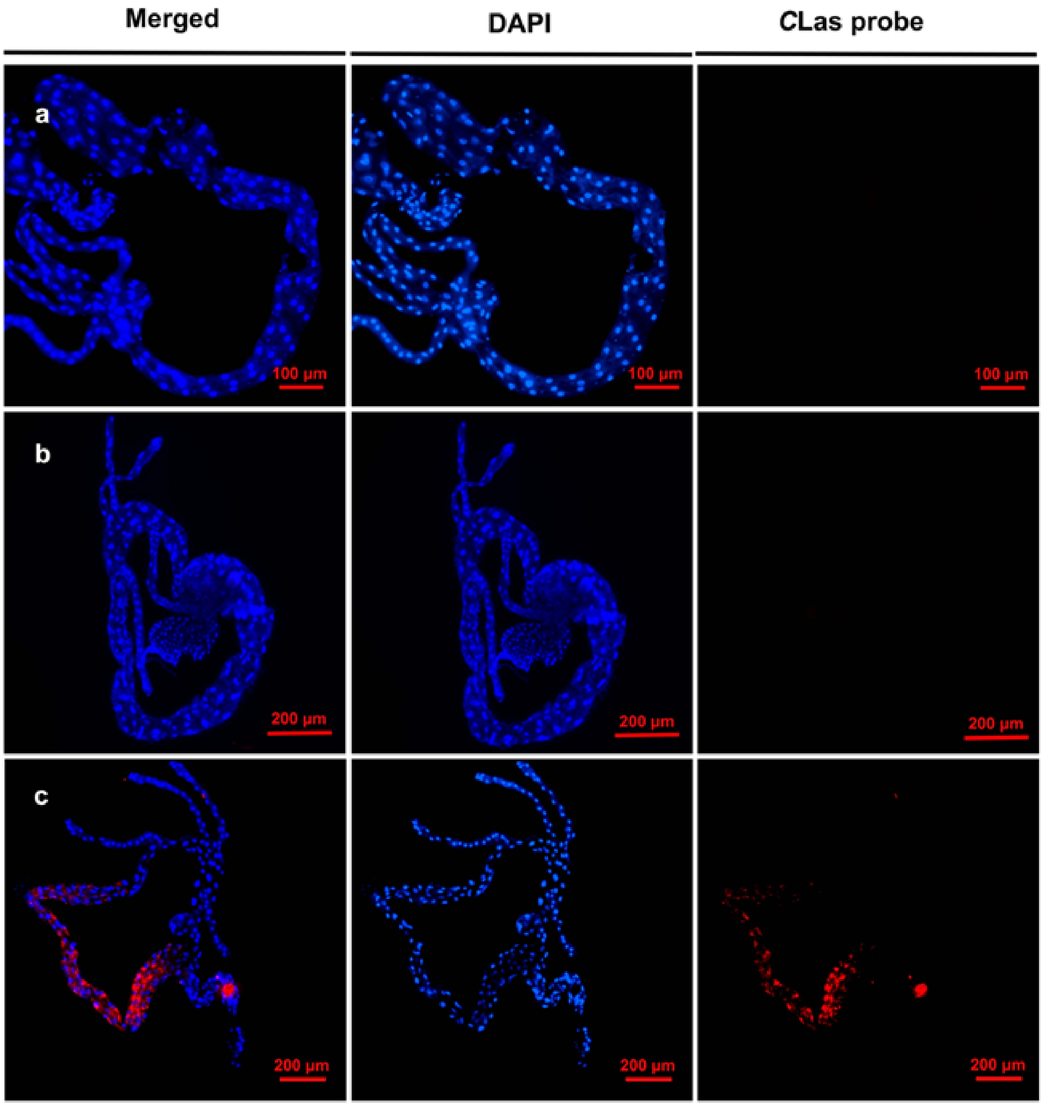

Supplementary Figure 3. FISH visualization of *C*Las in the midgut of *C*Las-recipient Asian citrus psyllid.

a: midgut of 5th instar ACP nymphs; b: midgut of 8-day age ACP adults; c: midgut of plant-vectored *C*Las positive ACP adults. The left column shows merged images, the middle column shows the DAPI staining (blue) and the right column shows the *C*Las probe (red) images.

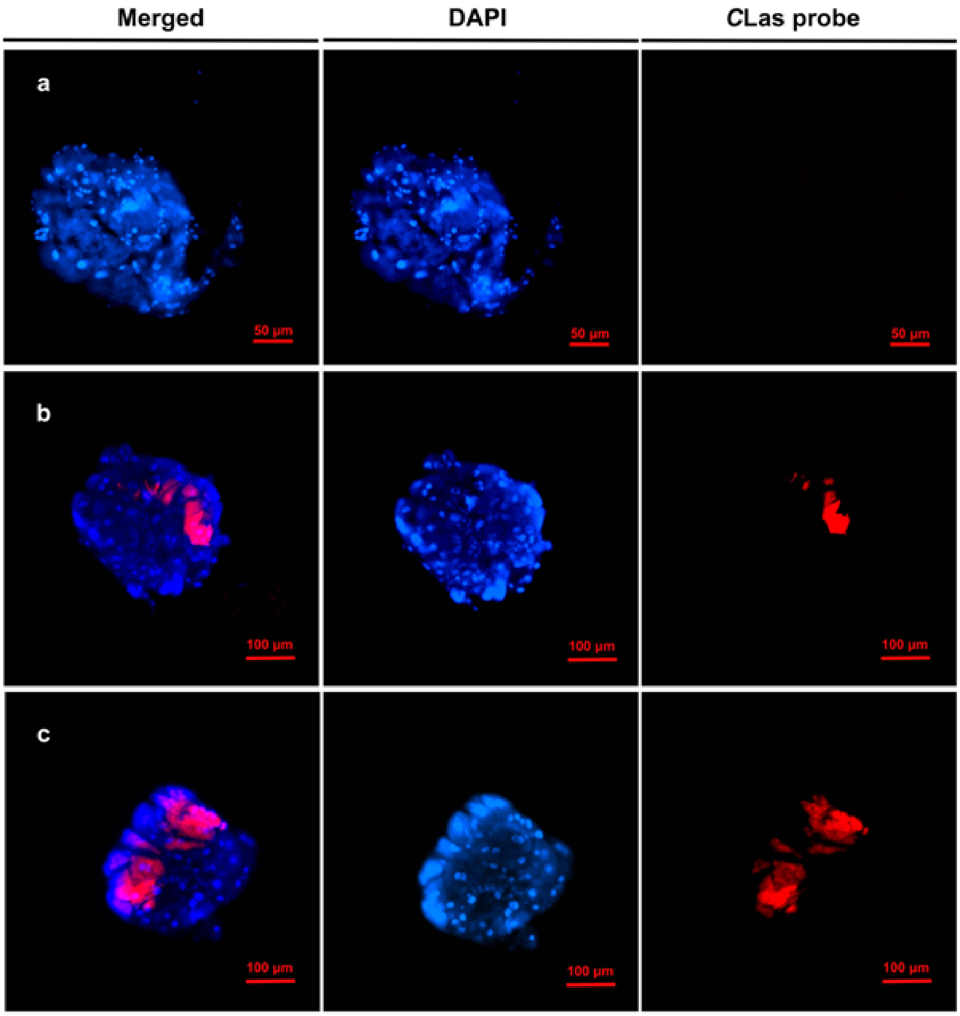

Supplementary Figure 4. FISH visualization of *C*Las in salivary glands of *C*Las-recipient Asian citrus psyllid. a: salivary glands of 5th instar ACP nymphs; b: salivary glands of 8-day ACP adults; c: salivary glands of plant-vectored *C*Las positive ACP adults. The left column shows merged images, the middle column shows the DAPI staining (blue), and the right column shows the *C*Las probe (red) images.

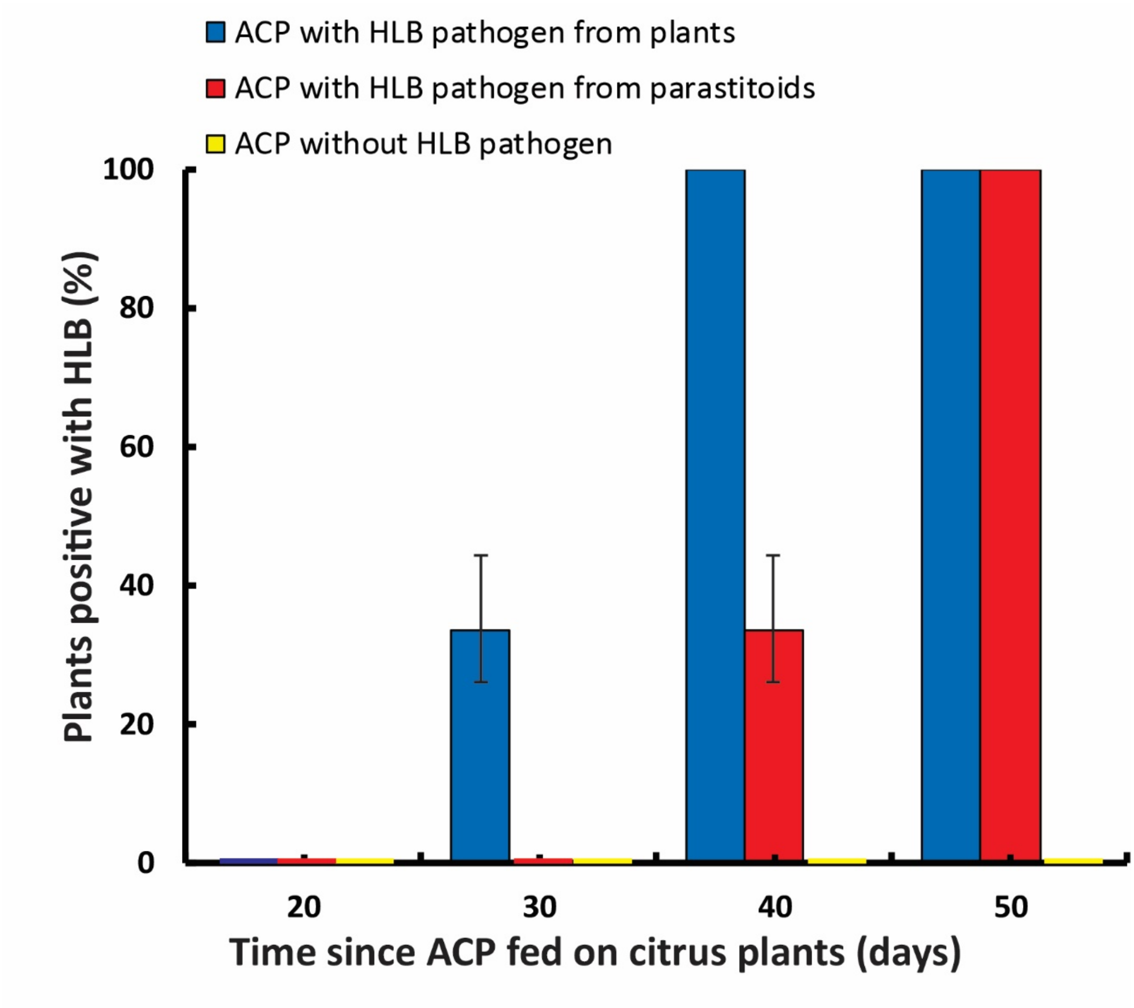

Supplementary Figure 5. *C*Las transmission efficiency from *Tamarixia radiata*-inoculated ACP to citrus plants.

*C*Las could not be positively detected in the citrus leaves that were fed on by *T. radiata*-inoculated ACP adults for 30 days; while the proportions of *C*Las positive citrus leaves increased to 33.3% and 100% after feeding on them by these ACP adults for 40 and 50 days, respectively. The error bars represent mean ±SE.
