# Supplementary material for: Parasitoid vectors a plant pathogen, potentially diminishing the benefits it confers as a biological control agent
Source: Commun Biol. 2021 Nov 25;4:1331. doi: 10.1038/s42003-021-02851-2 (PMC8617049; doi:10.1038/s42003-021-02851-2)
Supplement: Supplementary file 3 — Description of Additional Supplementary Files [file 42003_2021_2851_MOESM3_ESM.pdf]

Description of Additional Supplementary Files

File name: Supplementary Data 1
Description: We have uploaded all source data underlying the graphs and charts presented in the main figures.
