# Supplementary material for: Parasitoid vectors a plant pathogen, potentially diminishing the benefits it confers as a biological control agent
Source: Commun Biol. 2021 Nov 25;4:1331. doi: 10.1038/s42003-021-02851-2 (PMC8617049; doi:10.1038/s42003-021-02851-2)
Supplement: Supplementary file 4 — Supplementary Data 1 [file 42003_2021_2851_MOESM4_ESM.docx]

**Supplementary Data 1: Raw data of the *C*Las quantitative analysis in different experiments**

**Figure 1.** Acquisition and persistence of *C*Las in *Tamarixia radiata* when they developed from *C*Las donor ACP hosts

**A: Relative titers of *C*Las in different stages of *T. radiata***

| **Relative titers**  **of *C*Las** | egg | 1st | 2nd | 3rd | 4th | Pupa | Adult |
| --- | --- | --- | --- | --- | --- | --- | --- |
| Replicate 1 | 0.1267 | 1.6133 | 2.0849 | 15.1369 | 52.7098 | 0.4863 | 1.0281 |
| Replicate 2 | 0.1340 | 1.2483 | 3.7064 | 18.7654 | 48.8403 | 0.5548 | 0.9593 |
| Replicate 3 | 0.1406 | 1.9588 | 2.0420 | 19.2929 | 64.0000 | 0.5510 | 1.0000 |

**B: Relative titers of *C*Las in female and male *T. radiata***

| **Relative titers of *C*Las** | Female | Male |
| --- | --- | --- |
| Replicate 1 | 1.0281 | 7.7275 |
| Replicate 2 | 0.9593 | 7.7275 |
| Replicate 3 | 1.0000 | 7.0128 |

**C: Relative titers of *C*Las in different tissues of *T. radiata***

| **Relative titers**  **of *C*Las** | Hem | Gut | FB | Ov | PS | SG | Sp | Mu | Hea |
| --- | --- | --- | --- | --- | --- | --- | --- | --- | --- |
| Replicate 1 | 14.7200 | 3.0700 | 2.7500 | 2.6900 | 1.0000 | 0.7700 | 0.8200 | 0.2500 | 0.0200 |
| Replicate 2 | 16.6800 | 2.9900 | 2.3800 | 2.2500 | 1.0000 | 0.9000 | 0.7800 | 0.1800 | 0.0200 |
| Replicate 3 | 15.3500 | 2.7700 | 2.4300 | 1.9900 | 1.0000 | 0.5900 | 0.6500 | 0.1300 | 0.0200 |

**D: Relative titers of *C*Las at different days after eclosion**

| **Relative titers of *C*Las** | 1d | 5d | 10d | 15d |
| --- | --- | --- | --- | --- |
| Replicate 1 | 1.2953 | 0.4424 | 0.0000 | 0.0000 |
| Replicate 2 | 0.9615 | 0.2980 | 0.0000 | 0.0000 |
| Replicate 3 | 0.8029 | 0.4099 | 0.0000 | 0.0000 |

**Figure 4. Relative titers of *C*Las in the *Tamarixia radiata* F_1_ generation.**

| **Relative titers of *C*Las** | Egg | 1st | 2nd | 3rd | 4th | Pupa | Adult |
| --- | --- | --- | --- | --- | --- | --- | --- |
| Replicate 1 | 1.0792 | 0.0352 | 0.0190 | 0.0115 | 0.0122 | 0.0000 | 0.0000 |
| Replicate 2 | 0.9266 | 0.0461 | 0.0133 | 0.0091 | 0.0146 | 0.0000 | 0.0000 |
| Replicate 3 | 0.9862 | 0.0458 | 0.0120 | 0.0089 | 0.0123 | 0.0000 | 0.0000 |

**Figure 6. The relative titers of *C*Las in the 5^th^ instar nymphs and adults of *C*Las-recipient Asian citrus psyllid.**

| **Relative titers of *C*Las** | FI-HEM | AD-HEM | FI-SG | AD-SG | FI-GUT | AD-GUT |
| --- | --- | --- | --- | --- | --- | --- |
| Replicate 1 | 0.2736 | 1.1810 | 0.0000 | 4.6913 | 0.0000 | 0.0000 |
| Replicate 2 | 0.2994 | 0.8645 | 0.0000 | 2.2658 | 0.0000 | 0.0000 |
| Replicate 3 | 0.2932 | 0.9794 | 0.0000 | 3.1383 | 0.0000 | 0.0000 |

**Figure S5 *C*Las transmission efficiency from *C*Las-recipient ACP to citrus plants.**

| **Days since ACP feeding on citrus plants(d)** | Replicate | **Percent of plants positive with HLB (%)** | | |
| --- | --- | --- | --- | --- |
|  |  | ACP with HLB pathogen from plants | ACP with HLB pathogen from parasitoids | ACP without HLB pathogen |
| 20d | Replicate 1 | 0 | 0 | 0 |
|  | Replicate 2 | 0 | 0 | 0 |
|  | Replicate 3 | 0 | 0 | 0 |
| 30d | Replicate 1 | 33.33 | 0 | 0 |
|  | Replicate 2 | 16.67 | 0 | 0 |
|  | Replicate 3 | 50 | 0 | 0 |
| 40d | Replicate 1 | 100 | 16.67 | 0 |
|  | Replicate 2 | 100 | 33.33 | 0 |
|  | Replicate 3 | 100 | 50 | 0 |
| 50d | Replicate 1 | 100 | 100 | 0 |
|  | Replicate 2 | 100 | 100 | 0 |
|  | Replicate 3 | 100 | 100 | 0 |
